# Mice with Alopecia, Osteoporosis, and Systemic Amyloidosis Due to Mutation in *Zdhhc13*, a Gene Coding for Palmitoyl Acyltransferase

**DOI:** 10.1371/journal.pgen.1000985

**Published:** 2010-06-10

**Authors:** Amir N. Saleem, Yen-Hui Chen, Hwa Jin Baek, Ya-Wen Hsiao, Hong-Wen Huang, Hsiao-Jung Kao, Kai-Ming Liu, Li-Fen Shen, I-wen Song, Chen-Pei D. Tu, Jer-Yuarn Wu, Tateki Kikuchi, Monica J. Justice, Jeffrey J. Y. Yen, Yuan-Tsong Chen

**Affiliations:** 1 Institute of Biomedical Sciences, Academia Sinica, Nankang, Taiwan; 2 Department of Internal and Preventive Medicine, College of Veterinary Medicine, University of Mosul, Mosul, Iraq; 3 Taiwan Mouse Clinic–National Phenotyping Center, National Research Program for Genomic Medicine, National Science Council, Taipei, Taiwan; 4 Department of Molecular and Human Genetics, Baylor College of Medicine, Houston, Texas, United States of America; 5 Department of Biochemistry and Molecular Biology, Pennsylvania State University, University Park, Pennsylvania, United States of America; 6 Department of Pediatrics, Duke University Medical Center, Durham, North Carolina, United States of America; Massachusetts General Hospital, United States of America

## Abstract

Protein palmitoylation has emerged as an important mechanism for regulating protein trafficking, stability, and protein–protein interactions; however, its relevance to disease processes is not clear. Using a genome-wide, phenotype driven *N*-ethyl-*N*-nitrosourea–mediated mutagenesis screen, we identified mice with failure to thrive, shortened life span, skin and hair abnormalities including alopecia, severe osteoporosis, and systemic amyloidosis (both AA and AL amyloids depositions). Whole-genome homozygosity mapping with 295 SNP markers and fine mapping with an additional 50 SNPs localized the disease gene to chromosome 7 between 53.9 and 56.3 Mb. A nonsense mutation (c.1273A>T) was located in exon 12 of the *Zdhhc13* gene (Zinc finger, DHHC domain containing 13), a gene coding for palmitoyl transferase. The mutation predicted a truncated protein (R425X), and real-time PCR showed markedly reduced *Zdhhc13* mRNA. A second gene trap allele of *Zdhhc13* has the same phenotypes, suggesting that this is a loss of function allele. This is the first report that palmitoyl transferase deficiency causes a severe phenotype, and it establishes a direct link between protein palmitoylation and regulation of diverse physiologic functions where its absence can result in profound disease pathology. This mouse model can be used to investigate mechanisms where improper palmitoylation leads to disease processes and to understand molecular mechanisms underlying human alopecia, osteoporosis, and amyloidosis and many other neurodegenerative diseases caused by protein misfolding and amyloidosis.

## Author Summary

Palmitoylation, the addition of palmitate (a fatty acid) to protein, is one of the most common post-translational lipid modifications and has recently emerged as an important mechanism for modulating protein targeting, trafficking, stability, and protein–protein interactions. However, its physiological role and its relevance to the disease processes are not at all clear. Here we reported that mice with mutation in *Zdhhc13*, a gene coding for palmitoyl acyltransferase that catalyzes the reaction of protein palmitoylation, exhibited a severe phenotype and profound pathology involving multi-organ/systems. These mice showed wasting, weight loss, hair loss (alopecia), reduced bone mineral density (osteoporosis), and generalized amyloid deposition, which resulted in early death. Our results established a direct link between protein palmitoylation and regulation of the important diverse physiological functions and indicated that its absence can result in profound disease pathology. This mouse model will be useful for further understanding the molecular mechanisms underlying human alopecia, osteoporosis, and many other neurodegenerative diseases caused by protein misfolding and amyloid deposition.

## Introduction

Proteins can be modified by a variety of lipids, including myristate (C14), farnesyl (C15), palmitate (C16), geranylgeranyl (C20) and glycosylphosphatidylinositol (GPI). Palmitoylation is one of the most common post-translational lipid modifications that involve the addition of palmitate to specific cysteine residues of proteins via a thioester linkage [1]–[3]. Although most of the lipid modifications are irreversible, protein S-palmitoylation can be either permanent or transient, which allows it to dynamically regulate protein function [1],[4]. Numerous soluble and integral membrane proteins have been shown to be palmitoylated including signaling proteins, enzymes, scaffolding proteins, ion channels, cell adhesion molecules and neuronal proteins. Specific examples are oncogenic Ras proteins, trimeric G protein α subunit, Rap2b, RhoB, eNOS, SNAP-25, PSD-95 postsynaptic scaffolding protein, huntingtin and anthrax toxin receptor [1], [2], [5]–[8].

Palmitoyl post-translational modification has recently emerged as an important mechanism for modulating protein targeting, trafficking, stability and protein-protein interactions, and plays roles in numerous cellular processes, including signaling, apoptosis and neuronal transmission [1], [3].

Although palmitoylation was first described over 30 years ago, the genes coding for enzymes involved in protein palmitoylation, the palmitoyl acyltransferase (PATs), have only recently been discovered [7], [9]. To date, at least 23 members of PATs have been identified in the mammalian genome [9], [10]. This family of proteins contains a cysteine-rich domain (CRD) with a core Asp-His-His-Cys (DHHC) motif that is essential for PAT activity [7], [9], [11]. The presence of so many PATs in a single organism could be due to differences in substrate specificities, intracellular localizations or tissue distributions. For example, DHHC2 and DHHC15 are more specific to PSD-95 and GAP-43, DHHC9 and DHHC18 are specific to H-Ras and N-Ras, while DHHC3 and the closely related DHHC7 have broad substrate specificities [2], [8]. In neuronal tissue, DHHC13 and 17 modulate huntingtin palmitoylation and DHHC8 modulates paralemmin-1 [11]. The substrate specificity appears to be determined by the regulatory domains outside the DHHC domains of the enzymes [8], [11].

Despite the functional importance of protein palmitoylation at the cellular and biochemical levels, its physiological role and its relevance to disease processes is not clear. Oncogenic Ras proteins and huntingtin are direct targets for palmitoylation thus, they may be involved in the disease process. Disregulation of DHHC2 may be involved in cancer metastasis [12]. The sole mouse model of DHHC deficiency is the *Zdhhc8* knockout; these mice have a mild behavior phenotype with a decrease in exploratory activity and a deficiency in prepulse inhibition. These behavioral changes are only observed in female mice. The phenotypes together with genetic evidence may support the hypothesis that DHHC8 is a risk factor for schizophrenia [13]. Systematic knockdown of the *Zdhhc* genes has not been done, which could provide some unexpected physiological roles for DHHC proteins. Here we report on mice with a mutation in the *Zdhhc13*, a gene coding for palmitoyl acyltransferase, which catalyzes the reaction of protein palmitoylation [11]. Mutant mice exhibit a severe phenotype and profound pathology involving multi-organ/systems. These mice, developed cachexia, alopecia, osteoporosis, systemic amyloidosis, failed to thrive and succumbed to early death.

## Results

The mutant mice were analyzed either on a C57BL/6×129S6/SvEv or C57BL/6×129S6/SvEv × C3H mixed genetic background. The phenotypes described herein are all penetrant in both genetic backgrounds.

### Clinical Phenotypes

#### General appearance

**Figure 1:**
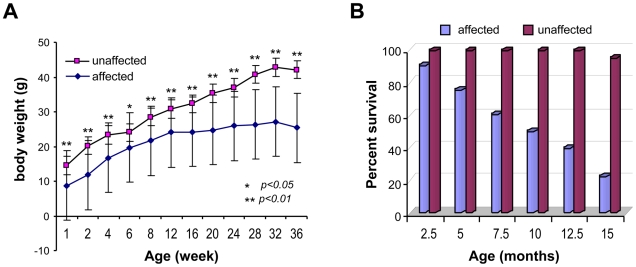
Body weight and life span of the affected mice. (A) Body weights of affected male mice compared to their unaffected male siblings (n = 10 each). Values are expressed as mean ± SE. (B) Life span of the affected mice compared to their unaffected siblings (n = 10 each).

**Figure 2:**
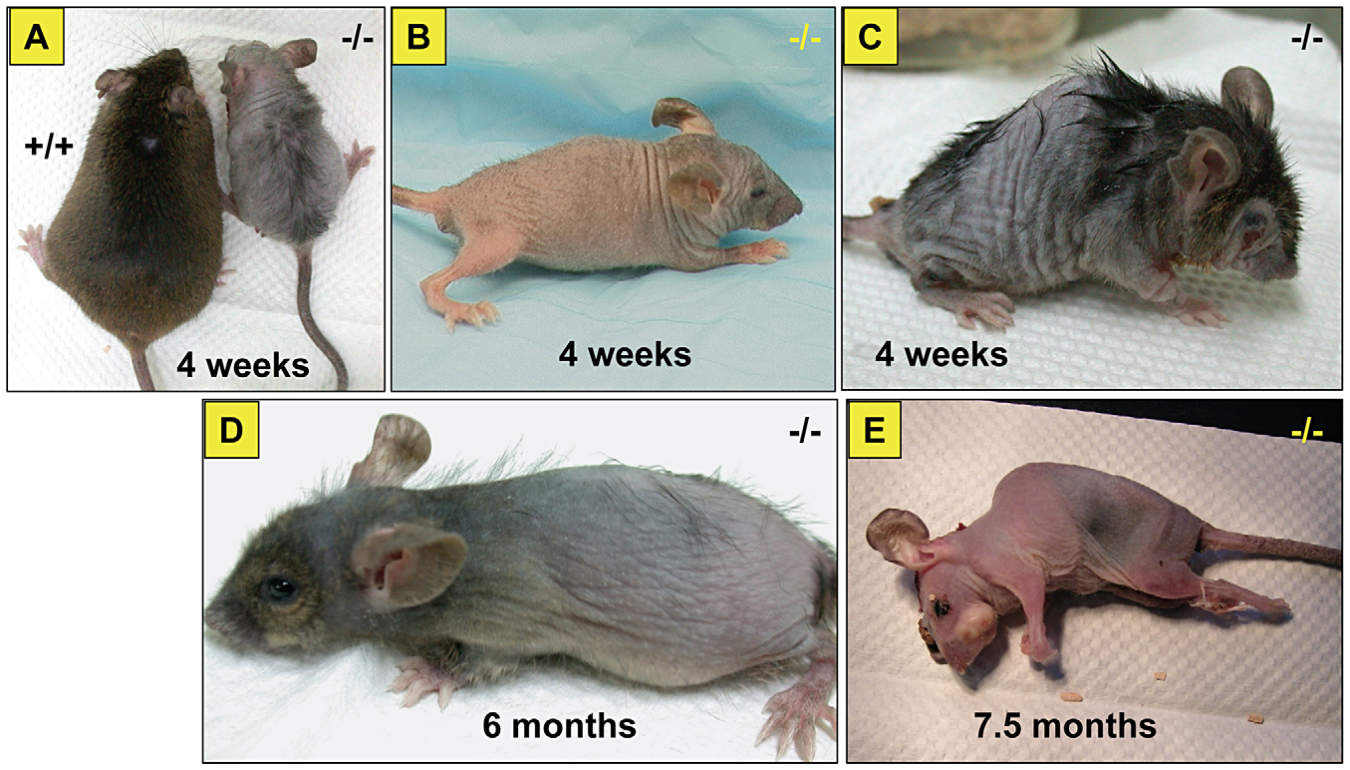
General appearance of the affected mice. Note the size difference of affected versus unaffected sibling (A). Affected mice at different ages, 4 weeks (B), kyphosis with sharper spine angle in affected mouse and patchy alopecia (C), at 6 months of age (D) and just before death at 7.5 months of age (E).

**Figure 3:**
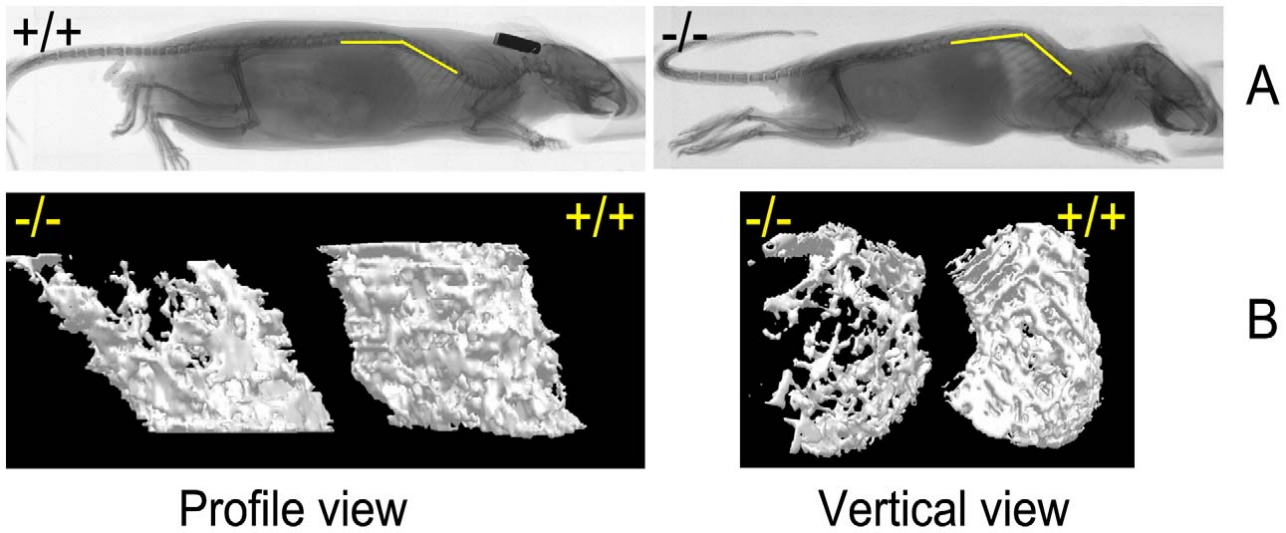
Skeletal abnormalities in the affected mice. (A) Radiographs of affected mouse and unaffected sibling at 26 weeks of age. Yellow bars indicate the position of spine. Scale bar = 1 cm. (B) Micro–CT imaging of the femur trabecular bone in the wild-type and skcm^04Jus^ mice taken at 26 weeks of age. The 3D images of trabecular bone were reconstructed as described in Materials and Methods; scale bar = 1 cm.

Affected mice appeared normal at birth, but by postnatal day 7 were small in size and developed hypotrichosis; these features differentiated affected from normal siblings. The affected male mice had poor weight gain and weighed 50% less than the unaffected siblings (Figure 1A). Affected females mice also showed similar poor weight gain (data not shown). In addition, these mice, regardless of sex, had a shortened life span; about 50% died before 7.5 months of age and only 20% survived beyond one year of age (Figure 1B). Furthermore, the affected mice showed generalized hypotrichosis and hair loss particularly certain body parts, some hairs remained over the head and back, although, they were thin, short and had decreased luster (Figure 2). Skin was loose with wrinkling and folding (Figure 2C). Kyphosis was evident beginning at day 28 (Figure 2, also shown in Figure 3A). When the gene responsible for these phenotypes was identified as *Zhddc13* (see below under Identification of the mutated gene), it was clear that only homozygous *Zdhhc13 -/-* exihibited abnormal phenotypes, while heterozygous *Zhddc13* +/− displayed normalcy like the wild-type (+/+).

#### Hematology and blood chemistry

Complete blood counts obtained from affected mice at 4 weeks and 30 weeks of age were comparable to the wild-type, except that adult mutant mice showed neutrophilia (mutant = 58.8±2.24 and unaffected = 38.5±4.13, *P<0.05*) and lymphocytopenia (mutant = 33.1±1.86 and 53.8±4.88 unaffected) despite normal WBC counts (Table S1). Blood biochemistry revealed elevations in AST and ALT enzymes in the adult (ALT in mutant = 100.6±15.3 and in unaffected = 52.8±5.07, AST in mutant = 44.5±7.18 and in unaffected = 24.8±2.72, *P<0.05*). However, at 4 weeks of age only AST was elevated (mutant = 101.6±15.6 and unaffected = 56.4±3.09, *P<0.05*) (Table S2). About 10% of adult mice also showed elevations in BUN (up to 180 mg/dl), CPK and total bilirubin. Serum calcium, magnesium and C-reactive protein were all within normal limits in the affected mice at both 4 weeks and 30 weeks of age.

#### Bone studies

Radiographic examinations showed severe kyphosis with marked increased spinal angle in the affected mice (Figure 3A). Osteoporosis was also profound, as evidenced by a decrease in the trabecular number of femur (Figure 3B and Table 1) and by other trabecular bone parameters, including a decrease in bone volume density (BV/TV) and bone mineral density (BMD), along with an increase in the structure model index (SMI), which indicated an abundance of rod-like trabeculae (Table 1). These features of osteoporosis could be seen as early as 4 weeks of age (data not shown).

**Table 1:** Structural parameters for trabecular bone.

Mouse genotype	Wild type (+/+)	Affected (−/−)
**BV/TV (%)**	9.58±3.22	2.96±1.21**
**Tb.Th (mm)**	0.07±0.01	0.07±0.002
**Tb.Sp (mm)**	0.44±0.03	0.6±0.11
**Tb.N (1/mm)**	1.37±0.33	0.45±0.18**
**SMI**	0.69±0.3	1.44±0.25**
**BMD (g/cm^3^)**	0.53±0.003	0.42±0.02**

**P<0.01 BV, trabecular bone TV, tissue volume; Tb.Th, trabecular thickness; Tb.Sp, trabecular separation; Tb.N, trabecular number; SMI, structure model index; BMD, bone mineral density.

#### Histopathological analyses

**Figure 4:**
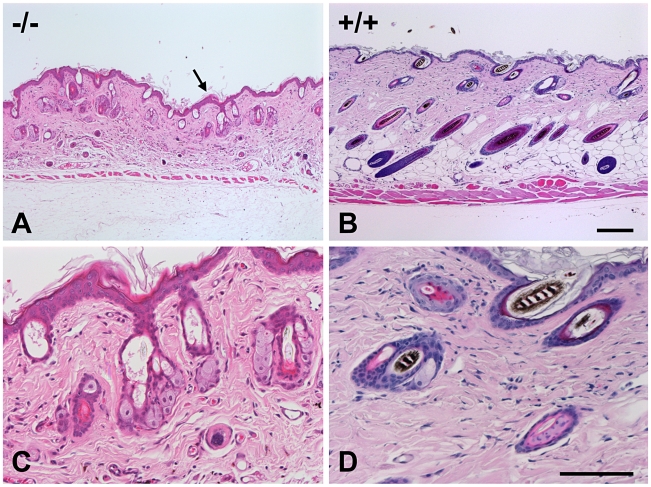
Skin histopathology of the affected mice. Skin of an affected mouse at age 16 weeks showed hyperkeratosis (arrow) and hyperplasia of the epidermis and thin dermis layer with scanty subcutaneous adipose tissue (A) when compared to a wild-type mouse (B). The hair follicles contained no hair shafts and their upper portions were dilated and filled with keratinized materials in a mutant mouse (C) as compared to the normal hair follicles in a wild-type mouse (D). (H&E, Bar = 200 µm in A and B; 100 µm in C and D).

**Figure 5:**
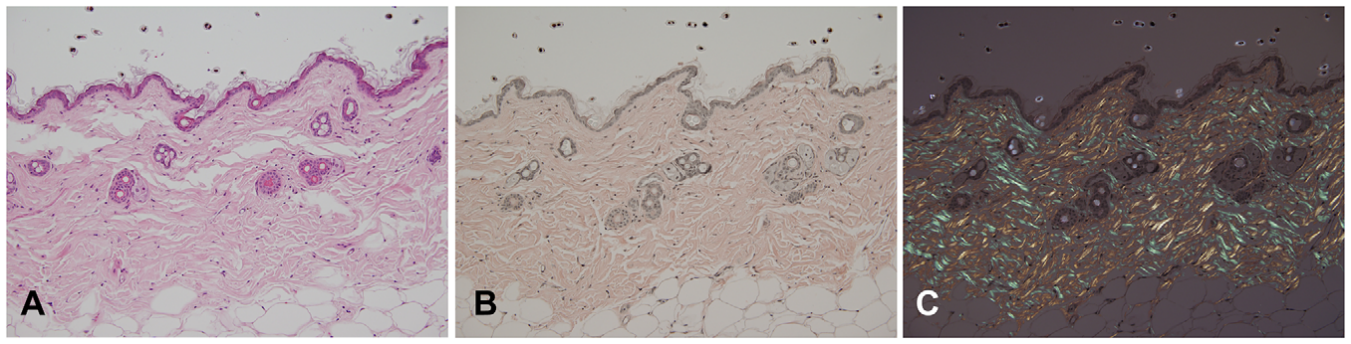
Histopathology analysis of amyloid in skin of an affected mouse. Skin sections were stained with H&E (A) and Congo red (B); and the latter stained section was also observed using a polarizing microscope (C). Note that amyloid deposits appeared eosinophilic by H&E stain, pink-red in Congo red stain, and showed yellow-green birefringence under a polarizing microscope.

Post mortem examinations revealed hepatosplenomegaly (2-3 times normal), severe muscle wasting and reduced white and brown adipose tissue. Skin histopathology of the affected mice showed hyperkeratosis and epidermis hyperplasia with thin dermis and scanty adipose tissue (Figure 4A). Hair follicles in different stages, such as anagen, catagen, and telogen, could be observed in wild-type mice (Figure 4D), while mutant mice had significantly fewer active hair follicles with most remaining in the late telogen phase. There were no hair shafts in the affected hair follicles and the upper portion was dilated (Figure 4C). Amyloid deposition was observed in the entire dermis in the affected mice as homogenous eosinophilic substances by H&E stain (Figure 5A). This was confirmed by Congo red stain as pink-red deposits (Figure 5B) and under a polarizing microscope as yellow-green birefringence (Figure 5C).

Amyloid depositions were also found in most of the other major organs examined, except for muscle. Amyloids were seen in liver, spleen, kidney, adrenal gland, pancreas, salivary glands, heart, lung, intestine and brain. The amyloids deposited in organs and their severity in young (4 weeks) and older animals (20–32 weeks) are summarized in Table 2. In general, there was a progressive increase of amyloid with age.

**Table 2:** Distributions and amyloid type in various tissues of the affected mice using Congo-Red Stain and immunohistochemistry.

Organ	Tissue	−/− age(24±1.85) (n = 10)*				−/− 4 weeks (n = 2)			
		CR	AA	ALλ	ALκ	CR	AA	ALλ	ALκ
**Liver**	Sinusoid	++	++	+	++	+	-	±	±
	portal vein	++	++	+	++	+	-	-	-
	central vein	++	±	-	±	-	-	-	-
**spleen**	red Pulp	+	+	+	+	+	-	±	±
	white Pulp	++	-	-	-	+	-	-	-
	central artery	++	±	±	±	-	-	-	-
**kidney**	glomerulus	++	+	+	++	+	+	+	+
	Tubules	++	+	+	++	+	-	±	-
	perivascular tissue	+	+	±	±	-	-	-	-
**Skin**	hair follicle	+	-	-	-	+	-	-	-
	dermis and epidermis	++	++	++	+	+	±	±	±
	subcutaneous tissue	+	+	+	+	-	±	±	±
**Adrenal gland**	Cortex	++	-	-	-	±	ND	ND	ND
	Medulla	++	++	-	++	-	ND	ND	ND
**Pancreas**	Acini	++	ND	ND	ND	±	ND	ND	ND
	Islets of Langerhans	++	ND	ND	ND	±	ND	ND	ND
	perivascular tissue	+	ND	ND	ND	±	ND	ND	ND
**Salivary gland**	acini cells	++	ND	ND	ND	+	ND	ND	ND
	blood vessel wall	++	ND	ND	ND	+	ND	ND	ND
**Heart**	cardiac myocyte	+	+	±	±	-	-	-	-
	perivascular tissue	++	+	±	+	-	-	-	-
**Lung**	Alveoli	+	ND	ND	ND	-	ND	ND	ND
	perivascular tissue	+	ND	ND	ND	-	ND	ND	ND
**Intestine**	Velli	+	±	±	±	-	-	-	-
	lamina properia	+	±	±	±	-	-	-	-
	perivascular tissue	+	±	±	±	-	-	-	-
**Brain**	Cerebellum	+	-	-	-	-	-	-	-
	perivascular tissue	+	-	-	-	-	-	-	-
	Cerebrum	±	-	-	-	-	-	-	-
**Bone**	bone marrow	±	ND	ND	ND	-	ND	ND	ND
**Muscle**	myofibril	-	ND	ND	ND	-	ND	ND	ND

(* *Zdhhc13^−/−^* adult mean age, (mean±SE) week, (- negative, ± Uncertainty + mild, ++ severe ND: not done).

**Figure 6:**
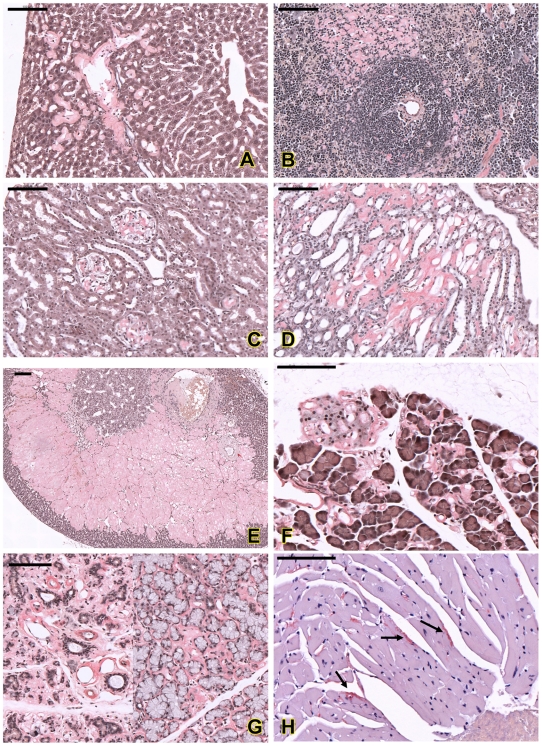
Histopathological analysis of amyloids in different organs of the affected mice. Amyloid deposition in sinusoids and around the portal vein in liver (A); in red pulp and peri-white pulp area in spleen (B). In kidney, amyloid was found in glomerulus (C) and renal tubules (D). Amyloid was also found in adrenal cortex and medulla (E), islet of Langerhans and around the acinar cells of pancreas (F), salivary glands (G) and myocardium (arrows in H). All sections were stained with Congo red; amyloid deposits appeared pink-red color with this staining. Bar = 100 µm.

In the liver, amyloid deposits were observed mainly in sinusoids around perivascular areas (Portal and central veins) (Figure 6A). Enlarged Kupffer cells containing amyloid substance were also seen and in severe cases, massive amyloid deposits disrupted the hepatic architecture (data not shown). Mild amyloid deposition in the space of Disse or sinusoid was also found in young mice as early as 4 weeks of age (Table 2).

In the spleen, accumulations of amyloid appeared in peri-white pulp, connective tissue frameworks of red pulp and central arterial walls of white pulp (Figure 6B). Mild amyloid deposition was also observed in young animals; however, the arterial wall findings were not detectable in young mutants (Table 2).

In the kidney, amyloid accumulated in the glomerulus (Figure 6C), renal tubules (Figure 6D) and perivascular areas. Thickenings of the basement membranes of glomerular capillaries and mesangial matrix were common lesions in mutants. In the advanced stage, the glomerular capillaries were obliterated, and the glomerular structures were completely destroyed. The renal tubules became dilated with large amounts of filtrated substances (as an eosinophilic substance, presumably albumin that is observed as a result of a glomerular filtration defect) and some tubular epithelial cells showed atrophy. In young animals, renal amyloidosis was milder, but could be seen in both glomerulus and tubules (Table 2).

Massive amyloid deposits were also observed in the adrenal glands in adult mice. Amyloid accumulated in the zona fasciculate, zona reticularis, and in part of the zona glomerulosa and medulla (Figure 6E).

In the pancreas, amyloid accumulated in both exocrine pancreatic tissues and islet of Langerhans (Figure 6F) and, in the advanced stage, acinar cells were degenerated and completely replaced by amyloid. Acinar cells in salivary glands were also full of amyloid (Figure 6G).

In the heart, amyloid deposition could be seen in the blood vessel walls, and only in severe cases, amyloid was observed in the myocardium (Figure 6H). Small amounts of amyloid deposits could also been found in other organs such as lung, intestine and brain. No amyloid deposition was found in skeletal muscle.

### Immunohistochemistry Staining

**Figure 7:**
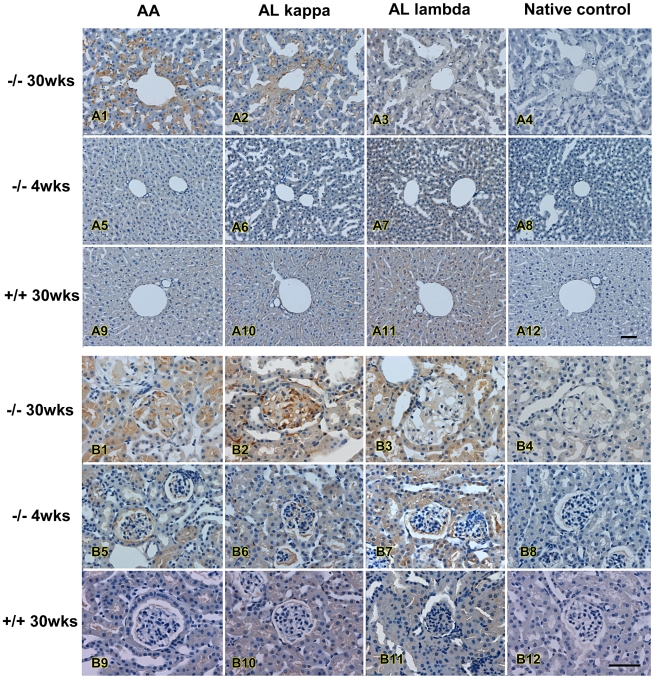
Immunohistochemistry of amyloidosis in affected mice. Immunohistochemistry analysis of amyloids in liver (upper panel) and in kidney (lower panel) of an affected mouse. Antibodies against AA amyloid (AA), κ light chain (ALκ), and λ light chain (AL λ) were used to differentiate types of amyloid; note progressively increase of amyloid deposition with age. Bar = 50 µm.

Immunohistochemistry staining was performed using anti-amyloid A, anti-kappa light chain and anti-lambda light chain to confirm the amyloidosis and to differentiate AA and AL type of amyloidosis. In liver, amyloid AA and AL κ were the major amyloid detected, primarily in sinusoids and around the portal vein, while the amount of AL λ amyloid was less (Figure 7, upper panel). A similar pattern was also observed in kidney glomerulus and tubular cells in which AA and AL κ were the predominant amyloids (Figure 7, lower panel). Both AA and AL κ could also be detected in the kidneys of the young animals (Table 2).

### Identification of the Mutant Gene

**Figure 8:**
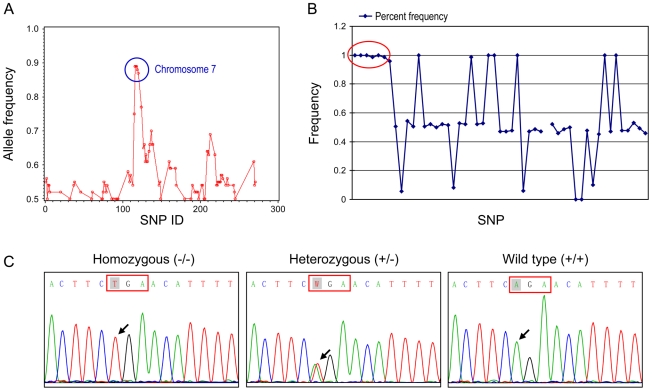
Mapping and molecular analyses of the gene responsible for the phenotypes. (A) Whole chromosomal mapping using 295 SNP markers. High homozygosity region is circled comprised of consecutive SNPs between SNP rs30814649 (46468726 bp) to SNP rs32491610 (64723695 bp) on chromosome 7. (B) Fine mapping of the candidate region using 52 SNPs on chromosome 7. Complete homozygosity is located between rs32116930 (53918742 bp) and rs32209625 (56317368 bp) (circled area). (C) DNA sequence analysis of mouse *Zdhhc13* gene. Nucleotide sequences in exon 12 showing that affected mouse was homozygous for T at position c.1273 (arrow); unaffected parent was heterozygous A/T and wild-type was A/A at the same position.

To map the gene responsible for these abnormal phenotypes, affected mice in the B6×129 mix background were out-crossed to C3H/HeJ mice to generate N1 (B6 and C3H hybrid) affected offspring, then were intercrossed. The offspring of this intercross (N1F1) were used in the genomic analysis. Whole genome SNP homozygosity mapping revealed one region located between 46.4 and 64.7 Mb (18.3 Mb) of chromosome seven with 90% B6 homozygosity in consecutive SNPs (Figure 8A). Fine mapping narrowed down the candidate region to within 2.4 Mb (between 53.9 and 56.3 Mb) on chromosome 7 (Figure 8B). This region contained 64 genes. Because our affected mice showed generalized amyloidosis, we concentrated on the amyloid related genes located in this region which included *Saa1l* (serum amyloid A-like 1), *Saa3* (serum amyloid A 3), *Saa4* (serum amyloid A 4), *Saa1* (serum amyloid A 1), *Saa2* (serum amyloid A 2) and *Zdhhc13* (zinc finger, DHHC domain containing 13). Direct DNA sequencing of genes in affected mice revealed a homozygous A to T substitution in exon 12 of *Zdhhc13* (c.1273A>T) (Figure 8C). The parents were heterozygous for this mutation. Further study showed that the homozygous c.1273A>T mutation (−/−) completely segregated with the abnormal phenotypes. Siblings, as well as parents, that were heterozygous for this mutation (+/−) were phenotypically normal. This A to T substitution resulted in a stop codon (AGA>TGA) (arg-425-stop codon) and predicted a truncated protein. No other mutations were found in the remaining exons of gene *Zdhhc13* or in its promoter region. All other amyloid-related genes in the candidate region were also normal without detectable mutations.

Real time RT-PCR showed that the tissue expression of *Zdhhc13* mRNA was significantly reduced to 26.23% in the liver and 15.59% in the kidney of the affected mice compared to the wild type, (Table 3). The decreased mRNA indicated nonsense RNA decay due to the premature stop codon in our mutant *Zdhhc13* mice.

**Table 3:** Real-time quantitative RT–PCR of *Zdhhc13* mRNA in mouse tissues.

Tissue	Liver		Kidney	
mRNA	Control ±SD	Affected ±SD	Control ±SD	Affected ±SD
Ct^1^ (*Zdhh13*)	24.75±0.11	27.15±0.18	23.2±0.24	25.11±0.21
Ct^1^ (*B-actin*)	20.55±0.39	20.85±0.16	20.43±0.34	19.66±0.21
ΔCt^2^	4.37±0.13	6.3±0.09	2.77±0.11	5.45±0.04
ΔΔCt^3^	0	1.93	0	2.68
2^(ΔΔCt)^ 4	100%	26.23%	100%	15.59%

**1** *Ct*, cycle threshold. Values represent triplicates of 3 wild type and 3 affected animals.

**2** Δ*Ct = Ct (Zdhhc13)- Ct (B-actin).*

**3** ΔΔ*Ct* = Δ*Ct* (Affected) - Δ*Ct* (Control).

**4** 2^(ΔΔ*Ct*)^ represents relative expression level of *Zdhhc13* in affected mouse tissues as compared to control.

**Figure 9:**
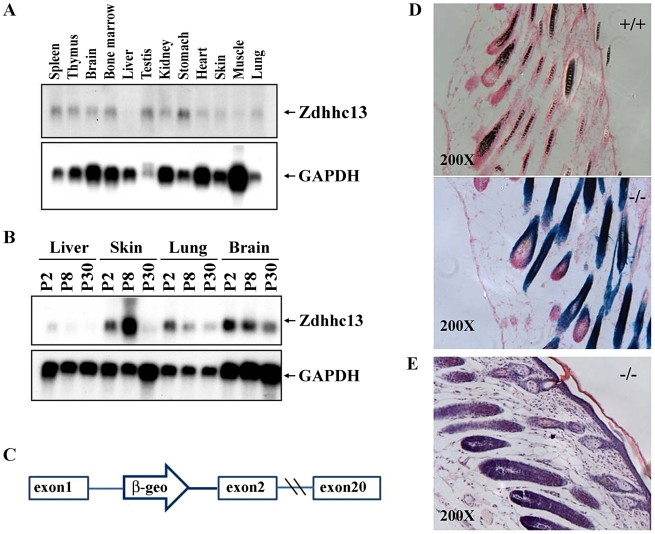
Expression of *Zdhhc13*. (A) Expression of *Zdhhc13* in normal adult tissues. GAPDH was used as a loading control, (B) Expression of *Zdhhc13* in liver, skin, lung and brain at postnatal (P) days 2, 8, and 30. Again, GAPDH was used as a control, (C) A gene trap vector insertion in intron 1 of *Zdhhc13*. The open arrow containing a β-geo cassette indicates the location of the gene trap vector, (D) Xgal staining of the p10 gene trap mutant and wild type. Note protein expression in the epithelium of the hair follicles. Sections were counterstained with nuclear fast red. The magnification is 200×, (E) Histopathology of gene traps mice skin, showing the abnormal follicles, lack of hair and thickened epidermis which are similar to ENU mutant mice.

To confirm that we had identified the correct gene, we obtained a gene trap allele in *Zdhhc13* from the SIGTR. This allele produced the same phenotype as the ENU-induced allele, with the exception of pink-eyes and a dilute coat color, which are associated with the closely linked *Oca2* locus carried in the 129/Ola gene trap ES cells. The skin which showed abnormal hair follicles, lack of hair and thickened epidermis (Figure 9E) which are similar to the mutant mice identified by the ENU mutation.

### Expression of *Zdhhc13*

We carried out Northern analysis of a variety of tissues to determine where *Zdhhc13* is expressed. We found that *Zdhhc13* is expressed in most adult tissues, but at low levels in the liver, skin, and lung (Figure 9A). We hypothesized that *Zdhhc13* may be needed most during development of these tissues, so we analyzed expression of *Zdhhc13* in liver, skin, lung and brain at three different time points: postnatal day (P) 2, P8 and P30. *Zdhhc13* was expressed most highly in the liver, lung, and brain at P2, showing that transcripts are developmentally regulated (Figure 9B). In contrast, *Zdhhc13* is expressed most highly in skin at P8, when hair follicles are maturing. We examined the gene trap allele, which contains a *lacZ* reporter gene, for expression of *Zdhhc13* in the skin, and found that it is expressed in the epithelium surrounding the hair follicles, consistent with a role in hair growth (Figure 9).

### Mutation in *Zdhhc13* Affects Protein Palmitoylation

**Figure 10:**
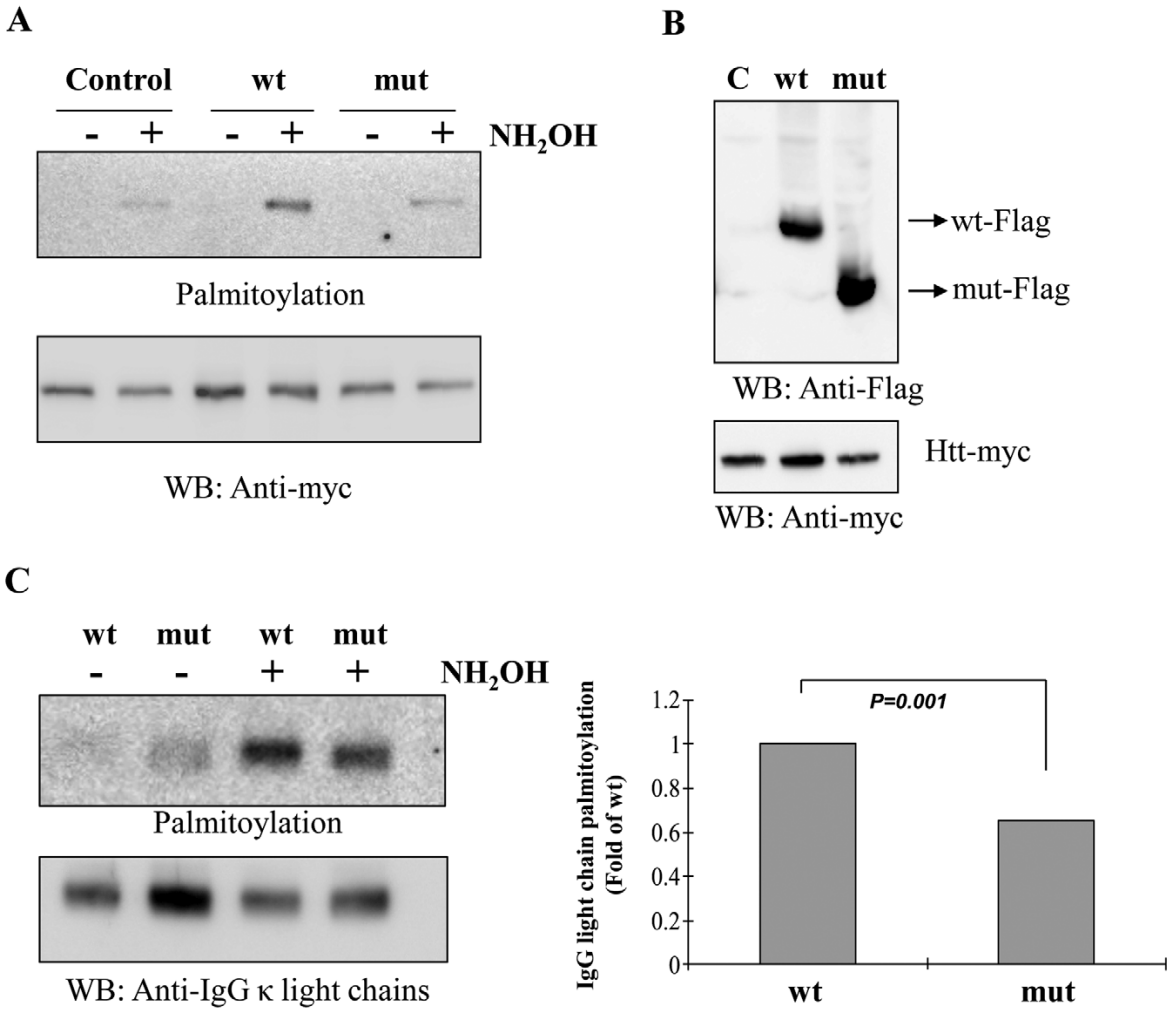
Palmitoyl-acyl transferase (PAT) activity and IgG light chain palmitoylation in the wild-type and mutant mice. (A) Acyl-biotin exchange assay showing palmitoylation of huntingtin (Htt) was greatly reduced by the mutant Zdhhc13 as compared to the wild type in the hydroxylamine (NH_2_OH)-treated group. Low panel was a loading control for huntingtin. Wt: wild *Zdhhc13*, Mut: mutant *Zdhhc13*, C: Control: cells transfected with huntingtin alone without co-transfection with Zdhhc13. WB: western blot, (B) HEK 293T cells co-transfected with huntingtin and Zdhhc13 showing expression of these proteins was approximately even, (C) Levels of IgG light chain palmitoylation in the wild-type and mutant mice. IgG light chain purified from serum of the wild and mutant mice were labeled with S-palmitoylation using acyl-biotin exchange method. IgG light chain treated with hydroxylamine showed reduced palmitoylated signals in mutant mice as compared to the wild type (p = 0.001, n = 3, t test). Low panel was a loading control.

Huntingtin is a known substrate of Zdhhc13 [11]. To demonstrate the palmitoylation defect caused by the mutation, HEK 293 T cells were co-transfected with huntingtin-myc and Zdhhc13-flag (WT and mutant). We examined the palmitoylation levels using acyl-biotin exchange assay after immunoprecipitation of huntingtin with anti-myc antibody and found that the huntingtin was palmitoylated by the wild-type Zdhhc13. The ability of mutant Zdhhc13 to palmitoylate huntingtin was greatly reduced by the mutant Zdhhc13, to a level indistinguishable from the endogenous palmitoyl activity present in the control (Figure 10A).

Moreover, when we examined the IgG light chain purified from serum of wild and mutant mice, we observed much less palmitoylated signals in the mutant mice as compared to the wild type mice (Figure 10C).

## Discussion

Using ENU-mutagenesis, we have identified mice with severe phenotypes manifested by failure to thrive, alopecia, osteoporosis, systemic amyloidosis and early death. We found that a nonsense mutation (R425X) in *Zdhhc13* was the cause of these abnormal phenotypes. To ensure that other mutations induced by ENU would not confound the observed phenotypes, mice examined after 6 generations of outcross breeding continued to show 100% phenotype and genotype correlations [14]. Further, a gene trap allele of *Zdhhc13*, which contains a vector inserted into the first intron, exhibits the similar phenotypes (data not shown).

*Zdhhc13*, also named huntingtin-interacting protein-like (HIP14L), shares 51% identity and 69% similarity with huntingtin-interacting protein-14 (HIP14) or *Zdhhc17* between #45 and #611 of the 622 residues [15]. Both *Zdhhc13* and *17* belong to a family of enzymes that are involved in attaching lipids to proteins, the palmitoyl acyltransferases (PATs). *Zdhhc13*, in addition to being a PAT, is also a mediator of Mg^2+^ transport. Inhibition of palmitoylation by 2-bromopalmitate (2BP) diminished Mg^2+^ transport by about 50% [16]. The causal mutation (R425X) in *Zdhhc13* mice would predict the synthesis of a truncated protein lacking the zinc-finger DHHC-CRD domain (#426-476) and the active site (C456) for the formation of an *S*-palmitoyl cysteine intermediate (Figure S1). Thus, it is unlikely that this truncated protein can perform any palmitoylation function, consistent with the similarity of phenotypes with the gene trap allele. Because our mutant mice had normal serum magnesium and calcium levels and demonstrated no clinical evidence of magnesium deficiency, we propose that the observed phenotypes originated from the loss of the enzymatic function of *Zdhhc13* as a PAT. Indeed, we have shown that mutation in the *Zdhhc13* affects the protein palmitoylation.

The exact mechanism by which the mutation of Z*dhhc13* resulted in such diverse pathologies is not clear. Amyloidosis is a devastating group of disorders in which normally soluble proteins are misfolded and aggregate to form insoluble amyloid fibrils with a β-sheet structure and presumably trigger an unfolded protein response (UPR) and its downstream pathways, including autophagy and cell death by apoptosis [17]-[19]. There are two major types of systemic amyloidosis. *AA-amyloidosis*, also called secondary or reactive amyloidosis, is a consequence of prolonged high level expression, mainly in the liver, of the acute-phase protein Serum Amyloid A precursor protein (SAA). *AA-amyloidosis* is usually associated with chronic inflammatory conditions but it can also be caused by mutations in a constitutively expressed protein, resulting in its greater tendency to aggregate [20], [21]. The second type of amyloidosis is called *AL-amyloidosis*. It is either a primary or a multiple myeloma-associated amyloidosis; its fibrils are derived from fragments of monoclonal immunoglobulin light chain (λ and κ) condensed into β-pleated sheet structures as a result of incomplete breakdown in the autophagolysosomes [22]. It is rare to find both AA and AL types in the same patient, and such cases only account for ∼2–3% of all amyloidosis patients [23].

Our *Zdhhc13* mutant mice manifested both AA and AL amyloids (Figure 5, Figure 6, Figure 7). Liver, spleen, kidneys, skin, adrenal and salivary glands are the most affected organs resulting in hepatosplenomegaly, nephromegaly, sialadenosis (non-inflammatory swelling of the salivary glands) and skin involvement. We cannot attribute this observed amyloidosis to inflammation, as CRP levels were normal (Table S2). Nor can we attribute it to multiple myeloma, as blood and bone marrow contained no excess plasma cells (data not shown). Furthermore, sequences of all amyloid-related genes in the candidate regions were normal except for the single mutation (R425X) in *Zdhhc13*. The coexistence of AL and AA systemic amyloidosis could be attributed to the fact that the presence of AL type amyloid fibrils acting as an amyloid-enhancing factor (AEF) and enhance the AA amyloid deposition [23]–[26]. Alternatively, a deficiency of *Zdhhc13* may play a role in the amyloidogenesis. Palmitoylation is known to affect protein stability by influencing a protein's access to an ubiquitinating enzyme [27]. Palmitoylation is also known to protect huntingtin from aggregation [28], and prevent oligomerization of certain proteins [29]. Moreover, defective palmitoylation results in aggregation of amyloid β-sheet, which leads to the formation of fibril [30].

Therefore, we propose that a deficiency of *Zdhhc13* PAT activity may have caused amyloidosis in our mice. The lack of *Zdhhc13* PAT activity affected palmitoylation of a set of unspecified protein targets and compromised their conformational stability and subcellular localization, eventually causing systemic amyloidosis of both the AA and AL types [26]. Coincidentally, a computer algorithm predicted the presence of palmitoylation site(s) on both SAA and the light chains of IgG [31]. Consistent with this notion was our demonstration of reduced level of palmitoylation of IgG light chain in the mutant mice.

Deficiency of protein palmitoylation in the *Zdhhc13* mutant mice could also explain the apparent osteoporosis because palmitoylation regulates osteoblast differentiation through bone morphogenesis protein (BMP)-induced *Osterix* expression [6]. Deletion of *Osterix* leads to a loss of mature osteoblasts and a lack of calcified bones (Osteoporosis), other signaling pathways such as NF-κB may be also involved in the development of the severe osteoporosis phenotype observed as early as weaning [32].

The *Zdhhc13* mutant mice also showed significant skin pathology with hypotrichosis, alopecia and loose skin with wrinkling and folding. Histopathology revealed epidermal hyperplasia with a thin dermis, inactive hair follicles and amyloid deposition. Expression analysis shows that expression of *Zdhhc13* is upregulated at the time of follicle maturation (P8), consistent with a direct role of *Zdhhc13* in hair formation. Although the exact molecular mechanisms are not clear, these phenotypes are consistent with defects in the NF-κB signaling pathways [33]. Another possible mechanism responsible for the skin pathology is the BMP-induced MAPK pathway as protein palmitoylation plays an important role in BMP-induced MAPK pathway activation [6]. BMP is involved not only in osteoblast differentiation but also in epidermal proliferation and differentiation, hair follicle cycling and innervations [34]. Since *Zdhhc13* normally upregulates both NF-κB and MAPK signaling pathways [35] the *Zdhhc13* mutation may significantly affect these pathways, leading to the disease phenotypes.

In summary, we report that deficiency of a single palmitoyl acyltransferase (*Zdhhc13*) can cause severe systemic phenotypes, including failure to thrive, cachexia, osteoporosis, alopecia, multi-organs/systems dysfunction secondary to systemic amyloidosis and early death. Our results established a direct link between protein palmitoylation and regulation of important diverse physiological functions and indicated that its absence can result in profound disease pathology. This mouse model will be useful for further investigation of the mechanisms by which improper palmitoylation leads to disease processes. The identification of target proteins of *ZDHHC13* would be an important first step for understanding the molecular mechanisms underlying human alopecia, osteoporosis and many neurodegenerative diseases caused by protein misfolding and amyloidosis.

## Materials and Methods

### Mouse Lines

The first recessive mutant allele was generated by a conventional ENU mutagenesis regimen. [36]. Briefly, multiple doses of *N*-ethyl-*N*-nitrosourea (ENU) (100 mg per kg body weight) were injected to mutagenize spermatogonia of C57BL/6J males (G0 generation). Recessive mutations were isolated in the third generation of breeding to females that carried either the balancer chromosome *129S6.Inv(11)8Brd* or *129.Rex* mutations, both of which had been made congenic on a 129S6/SvEvTac genetic background (N = 10). The mutant mice reported here were identified by their small size and hypotrichosis as early as postnatal day 7 and were designated as skin and coat mutation 4 (*skcm^04Jus^*). The mutant line was inherited as a recessive trait that segregated independently of the chromosome 11 balancer and the phenotype was completely penetrant in the 129S6/SvEv genetic background, and, later on, in the C3H background. The experimental protocols in this study were reviewed and approved by the Institutional Animal Care and Utilization Committee of Academia Sinica.

The gene trap allele was produced from embryonic stem (ES) cells AC0492 obtained from the Sanger Institute Gene Trap Resource (SIGTR). ES cells were injected into C57BL/6J blastocysts by the Darwin Genetics Core at Baylor College of Medicine. Chimeras were obtained, mated to C57BL/6J mice, and the allele was transmitted through the germline to generate *Zdhhc13^SIGTR^* mice. Subsequent experiments were performed on mice from this mixed 129/B6 genetic background. After initial genotyping of ES cells for the gene trap allele per protocols available from the resource, the phenotype was used to follow transmission of the allele. *Zdhhc13* is located at 56 Mb on Chromosome 7, which is only 7 Mb from Oculocutaneous albinism 2 (*Oca2*; pink-eyed dilution), which is located at 63 Mb. Therefore, mice carrying the gene trap allele were also pink-eyed and had dilute coat colors because of the *Oca2* mutation carried in the 129/Ola ES cells used to generate the gene trap.

### Blood Chemistry

Blood samples were obtained through an incision of the tail artery or by cardiac puncture at the time of sacrifice and collected in a heparinized tube (MICROTAINER, BD Diagnostics, Franklin Lakes, NJ). Complete hemogram was carried out using Abbott Cell-DYN 3700 Veterinary Haematology Analyzer (Abbott Laboratory, Illinois, USA). Thin blood smears were taken directly from the tail artery, fixed with absolute methanol for 5 minutes and stained by modified Wright's Giemsa stain for Plasma cells (Plasma B cells) identification. Plasma was analyzed using the FUJI DRI-CHEM SYSTEM 3500s (Fuji Photo Film Co. Ltd.) for measurement of aspartate aminotransferase (AST; U/l), alanine aminotransferase (ALT; U/l), creatinine phosphokinase (CPK; U/l), total cholesterol (TCHO; mg/dl), total protein (TP; g/dl), albumin (ALB; g/dl), globulin (GLO; g/dl), total bilirubin (TBIL; mg/d), blood urea nitrogen (BUN; mg/dl), C-reactive protein (CRP; mg/dl), calcium (Ca; g/dl) and magnesium (Mg; g/dl).

### Micro-Computed Tomography (Micro–CT) Analysis of Bone

For trabecular bone analysis and 3D images, a micro-CT scanner (Skyscan-1076, Skyscan, Belgium) was operated at 50 kV, 200 *uA*, 0.4° of rotation step, 0.5 mm Al filter and 9 um/pixel of scan resolution. For bone mineral density (BMD) analysis, it was operated at 50KV, 200 *uA*, 1° of rotation step, 0.5 mm Al filter and 35 um/pixel of scan resolution. Cross-sections were reconstructed using a cone-beam algorithm (software Cone_rec; Skyscan, Belgium). Files were then imported into CTAn software (Skyscan) for three-dimensional analysis and three-dimensional image generation. BMD for each femur was measured by CTAn, which was calibrated using of phantoms with known BMD (0.25∼0.75 g/cm^3^).

### Histopathology

Mice were sacrificed with overdoses of sodium pentobarbital for the histop-athological examinations. After flushing with normal saline, mice were perfused through the heart with 4% paraformaldehyde in 0.1M PBS, pH7.4, the perfusion flow rate, (4 ml/min) was controlled by an infusion pump (Bio-Rad, Econo Pump). A total of 37 organs and tissues, including heart, lung, liver, kidney, spleen, pancreas, adrenal gland, salivary gland, brain, skin, adipose tissues, skeletal muscles and bone, were removed, embedded in paraffin, cut into 5 µm sections and stained with hematoxylin-eosin (H&E) for general pathological examinations. Other serial sections were also processed for Congo Red staining to detect amyloid; and immunohistochemistry staining for amyloid classification. Bone marrow was aspirated from both femora for bone marrow smears immediately after sacrificing by cervical vertebral dislocation. Smears were fixed with absolute ethanol for 5 minutes and stained by modified Wright's Giemsa stain for Plasma cells identification.

### Immunohistochemistry

Immunostaining used the following antibodies: rabbit anti-human λ light chains polyclonal antibody, rabbit anti-human κ light chains polyclonal antibody, mouse anti-human amyloid A monoclonal antibody (DakoCytomation). Tissue sections were pretreated with concentrated formic acid for 1 min, washed in tris-buffered saline (TBS) for 10 min, then incubated in Rodent Block M (BioCare) or tris-buffered saline Tween (TBST) containing 2% bovine serum albumin and 3% normal goat serum at 37°C for 30 min. Then, tissue sections were incubated with primary antibody, washed in TBST, incubated in 3% H_2_O_2_ for 15 min at room temperature and then washed again in TBST followed by incubation with horseradish peroxidase-conjugated secondary antibody (anti-rabbit IgG and goat anti-mouse IgG (Jackson ImmunoResearch. West Grove, USA). Color was developed with 0.1% 3,3′-diaminobenzidine.

### Mapping of Gene Responsible for Abnormal Phenotypes

For the purpose of rough mapping, the affected *skcm^04Jus^* mice, which were in a mixed 129S6/SvEv and C57BL/6 mixed genetic background at the N = 4 generation on 129S6/SvEvTac (obtained from Baylor College of Medicine) were outcrossed to the C3He/HeJ strain in Academia Sinica to generate N1 offspring, and N1 mice were then intercrossed with generate N1F1 offspring. DNA was collected from 32 affected N1F1 mice. A panel of 295 single nucleotide polymorphism (SNP) markers located on all 19 mouse autosomes and the X-chromosome for mouse strains C3H/HeJ, C57BL/6, DBA/2J or BALB/cByJ was selected from a SNP dataset containing 10,915 SNPs from 48 mouse strains (provided by Tim Wiltshire, Genomics Institute of the Novartis Research Foundation, San Diego, California). SNPs were chosen based on the criterion that the genotype of these strains at the 295 loci was different from that of C57BL/6J. Since point mutations were introduced into C57BL/6J genome by ENU, the recessive mutant phenotype will always associated with a homozygous B6 SNP genotype at the mutant locus. SNP genotyping using genomic DNA isolated from mouse tails (Puregene DNA purification kit, Gentra Systems, Minneapolis, MN, USA) was performed using high-throughput MALDI-TOF mass spectrometry [37], [38]. Primers and probes flanking the SNPs were designed in multiplex format using SpectroDESIGNER software (Sequenom, San Diego, CA, USA). PCRs were performed in a volume of 5 µl containing 0.15 U of *Taq* polymerase (HotStarTaq, Qiagen, Valencia, CA, and USA), 5.0 ng of genomic DNA, 1.0 pmol of each PCR primer and 2.5 nmol of dNTP. Thermocycling conditions were one cycle at 94°C for 15 min, 45 cycles of 94°C for 20 s, 56°C for 30 s, 72°C for 30 s and one final cycle of extension at 72°C for 3 min. Unincorporated dNTPs were dephosphorylated using 0.3 U of Shrimp Alkaline Phosphatase (Hoffman-LaRoche, Basel, Switzerland) followed by primer extension using 9 pmol of each primer extension probe, 4.5 nmole of the appropriate dNTP/ddNTP combination, and 1.28 U of Thermosequenase (Amersham Pharmacia, Piscataway, NJ, USA). Reactions were cycled at 94°C for 2 min, followed by 55 cycles of 94°C for 5 s, 52°C for 5 s and 72°C for 5 s. Following the addition of a cation exchange resin (SpectroCLEAN, Sequenom) to remove residual salt from the reactions, 15 nl of the purified primer extension reaction was spotted onto a 384-element silicon chip preloaded with 3-hydroxypicoloinic acid matrix (SpectroCHIP, Sequenom), using the SpectroPOINT (Sequenom). SpectroCHIPs were analyzed using a Bruker Biflex III MALDI-TOF SpectroREADER mass spectrometer (Sequenom) and spectra processed with SpectroTYPER (Sequenom).

For fine mapping, 52 SNPs covering the candidate region from 46468726 bp (SNP rs30814649) to 64723695 bp (SNP rs32491610) on chromosome 7 (Mouse Genomic Informatics (MGI) http://www.informatics.jax.org/javawi2/servlet/WIFetch?page=snpQF were selected. Strain C57BL/6 was used as a selected strain and C3He/HeJ and 129/SvEv as reference strains. DNA samples from 84 affected N1F1 mice and 10 parental heterozygous mice in a 96 well plate (MicroAmp Optical 96-Well Reaction Plate, *Applied Biosystems*) were used for SNP genotyping using high-throughput MALDI-TOF mass spectrometry.

### Identification of the Mutant Gene

All exons, exon-intron junctions and 2.5 kb promoter regions of candidate genes, *Saa11, Saa3, Saa4, Saa1, Saa2* and *Zdhhc13*, were amplified and sequenced. Primers were designed using the Primer3 program http://biotools.umassmed.edu/bioapps/primer3_www.cgi. The primers used for the detection of an exon 12 mutation in the *Zdhhc13* gene were F, 5′-CTGGGTTGAGAGTATTCCACA-3′ and R, 5′-GAGATTAGCCACAGAGCTTCG-3′. PCR reactions were performed in a final volume of 25 *µl*, containing 50 pmol of each primer (0.5 *ul*), 10× Taq Buffer (10 mM Tris–HCl (pH 8.3), 50 mM KCl) with 1.5 mM MgCl_2_ (2.5 *ul*), 2.5 mM dNTPs (2.5 *ul*) and *Taq* DNA polymerase (5 U/*ul*) MDBio, Inc. (0.25 *ul*). Amplification conditions were an initial denaturation of 4 min. at 94°C, followed by 20 cycles of touchdown PCR in 30 s at 94°C, 30 s at 65°C (decrease 0.5°C per cycle), 40 s at 72°C; and a final 20 cycles in 30 s at 94°C, 30 s at 55°C, followed by 40 s at 72°C and then a final extension at 72°C for 5 min. All amplified PCR fragments were digested with shrimp alkaline phosphatase and *Exo*I to remove unincorporated primers and sequenced using the BigDye Terminator Cycle Sequencing Kit v1.1/3.1 (Applied Biosystems, Foster City, CA, USA) following the manufacturer's instructions. Sequencing products were separated on either ABI PRISM 3100 Genetic Analyzer or ABI PRISM 3700 DNA Analyzer (Applied Biosystems). Raw sequencing data were analyzed with the DNA Sequencing Analysis Software v3.7 (Applied Biosystems).

### Expression Analysis

To examine the differences in the tissue expression of *Zdhhc13*, total RNA samples were extracted from liver and kidney of three 6-month old mutants as well as three aged-matched wild type C3He/HeJ mice using Trizol following the manufacturer's protocol. First-strand cDNA was synthesized using 1 µl oligo-dT 15 primer and 1 µl SuperScript III RT (200 U/1 µl) in 20 µl volume of 2 µg of total RNA, 1 µl of 10 mM dNTPs, 1 µl of reaction buffer (10 mM Tris-HCl pH 8.3, 2.5 mM KCl, 0.6 mM MgCl2), µl of RNase inhibitor40 U/µl, and 1 µl of 0.1 M DTT. Real-time quantitative RT-PCR analysis used the ABI PRISM 7700 Sequence Detection System (Applied Biosystems). RT-PCR amplification of *Zdhhc13* was carried out using the following primer set: 5′- GACTGGACGCTGCATAGGTT, forward strand in *Zdhhc13* exon13, and 5′- TGGCACAATGATTTGACCAG, reverse strand in *Zdhhc13* exon 15. The primers were designed using Primer Express (Applied Biosystems).

The cDNA corresponding to 75 ng of reversed transcribed total RNA was amplified in a final volume of 20*µl* using Power SYBER green PCR Master mix in 20*µl* total reaction volume in duplicate assays for *Zdhhc13* and endogenous *B-actin* as an internal control.

An analysis of the results was based on the *Ct* calculation, where *Ct* represents the cycle number at which fluorescence of the PCR samples crossed a given threshold. The expression level of β-actin was taken as the first “calibrator” to normalize the total *Zdhhc13* mRNA in each tissue (Δ*Ct*). Expression of, *Zdhhc13* in each of the control mouse tissue was then taken as the second “calibrator” to normalize the expression of *Zdhhc13* in the affected tissue accordingly (ΔΔ*Ct*). Final results were given as the relative amounts of *Zdhhc13* mRNA in the affected mouse tissues as compared to the control (2^ΔΔ*Ct*^).

Northern analysis was carried out as previously described by Lorenzetti *et al.* [39], using 10 ug of total RNA isolated using RNA STAT-60 reagent (TEL-TEST, Inc., Friendswood, TX) according to the manufacturer's protocol, and transferred to a nylon membrane. The blot was hybridized using Ultrahyb (Ambion) with a probe for *Zdhhc13* is the N-terminal 571 base pairs, which was PCR amplified with two primers (F-5′-ATGGAGGGCCCGGGCCT-3′, R-5′-TAAGCCGATAGCATGAGCG-3′). The probe for GAPDH is the N-terminal 509 base pairs, which was PCR amplified with two primers (F-5′-GGTCGGTGTGAACGGATTTGG-3′, R-5′-CATGAGCCCTTCCACAATGCC-3′). [39] B-galactosidase staining was carried out using X-gal staining, the skins of p1, p6 and p10 mice were embedded in Tissue Tek and frozen after fixation with 4% paraformaldehyde and sucrose protection. The 10 mm vertical cryosections were fixed again in 2% paraformaldehyde followed by serial washing (three times washing with 2 mM MgCl_2_ containing PBS, 0.02% NP40 and 0.01% deoxycholate). The sections were pre-incubated with the staining buffer (PBS supplemented with 5 mM K_3_Fe(CN)_6_, 5 mM K_3_Fe(CN)_6_, 2 mM MgCl_2_) for 2 min, and further incubated with the staining buffer supplemented with 1 mg/ml Xgal for 3 hrs. After washing with PBS, the sections were counterstained with Nuclear Fast Red (Vector) and then mounted.

### Evaluation of Enzyme Activity of Zdhhc13

We used huntingtin, a known substrate for Zdhhc13, and acyl-biotin exchange assay [11] to measure the palmitoyl acyl transferase activity. Plasmid construction: The cDNA of wild type and mutant Zdhhc13 were subcloned into C-terminal p3xFLAG-CMV vector (Sigma). Huntingtin (1–548 aa) cDNA was subscloned into pcDNA4/*myc*-His (Invitrogen). Cell culture: HEK293T cell were used for transiently transfected with Lipofectamine 2000 (Invitrogen). At 24–48 hours posttransfection, cells were harvested with PBS and proteins were extracted with lysis buffer (LB, 150 mM NaCl, 50 mM Tris-HCl, 5 mM EDTA, 1 *mM* PMSF, 1X protease inhibitor (Roche), 0.2% Triton X-100, pH 7.4) containing 50 mM N-ethylmaleimide (NEM) (Sigma). Immunoprecipitation (IP) and immunobloting (IB) of huntingtin protein and acyl-biotin exchange assay to label S-palmitoylated protein were performed using Myc antibody (Invitrogen), 1∶250 for IP; 1∶5000 for IB; and FLAG, mouse, antibody (Sigma) 1∶2000 for IB; as described previously [11].

### IgG Light Chains Purification and the Evaluation of S-Palmitoylation

Serum from 5 month old mice was used to purify the IgG light chain. Serum was incubated with Protein A/G agarose beads (Santa Cruze Biotechnology) for 1 hour at room temperature. After washing the beads with PBS for 3 times, IgG light chain were eluted with 0.2 *M* Glycine (pH 2.5) and neutralized with 1*M* Tris-HCl (pH 8.5). The eluted fractions were precipitated using the chloroform/methanol (C/M) precipitation method. Protein pellets were redissolved with 4% SDS buffer (50 *mM* Tris-HCl, 4% SDS, 5 *mM* EDTA, pH 7.4) containing 50 *mM* N-ethylmaleimide (NEM) (Sigma) for 10 minutes at 37°C. Following dilution with 3 *vol* of lysis buffer, the fraction was rotated end-over-end overnight at 4°C. Excess NEM was removed with 3 sequential C/M precipitations. Protein pellets were redissolved with 4% SDS buffer and divided to 2 portions. First portion was added to 3 *vol* of lysis buffer containing 1 *M* hydroxylamine, pH 7.2 and incubate for 1 hour at room temperature to remove palmitate group from the protein. Control portion was in lysis buffer without hydroxylamine. After incubation, 3 sequential C/M precipitations were performed on samples to remove hydroxylamine. Protein pellets were redissolved with 4% SDS buffer and were added with 3 *vol* of lysis buffer containing 0.5 *µM* biotin-BMCC (Pierce), pH 6.2 and incubate for 1 hour at 4°C to label the protein, followed by SDS-PAGE and Western blotting. Streptavidin protein- HRP (abcam) was used against biotinylated protein. Rabbit anti-human κ light chain polyclonal antibody (DakoCytomation) was used against IgG light chain.

## Supporting Information

Figure S1 Predicted secondary structure of *Zdhhc13* (Huntingtin-interacting protein 14-related protein, HIP14-related protein a palmitoyltransferase ZDHHC13). ZDHHC is 660 AA with molecular weight of 70890 Da, it has 6 transmembrane domains and 6 ANK repeats (ANK 1–6), Zn_Fing (DHHC-TYPE) length is 51aa (from 426–476aa) and the Bompbias (Phe-rich) is 64aa (from 328–391aa). The active site (S-palmitoyl cysteine intermediate) is located at residue 456. Note that nonsense mutation arg-425-stop codon is located before both Zn_Fing and the active site of the protein.
(0.10 MB TIF)

Table S1 Peripheral blood complete blood counts in affected and wild-type mice.
(0.05 MB RTF)

Table S2 Blood chemistry in affected and wild-type mice.
(0.05 MB RTF)
