# Supplementary figures and images for: Mice with Alopecia, Osteoporosis, and Systemic Amyloidosis Due to Mutation in Zdhhc13, a Gene Coding for Palmitoyl Acyltransferase
Source: PLoS Genet. 2010 Jun 10;6(6):e1000985. doi: 10.1371/journal.pgen.1000985 (PMC2883605; doi:10.1371/journal.pgen.1000985)

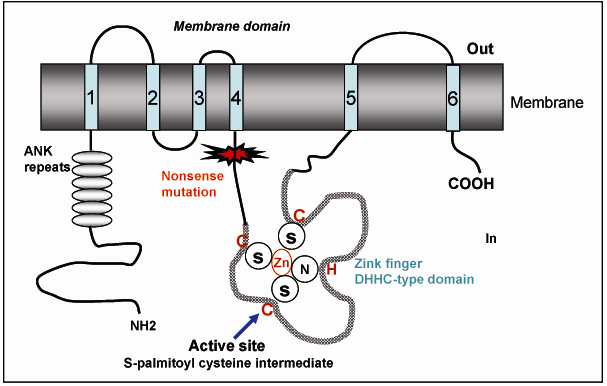

Supplement: Figure S1 — Predicted secondary structure of Zdhhc13 (Huntingtin-interacting protein 14-related protein, HIP14-related protein a palmitoyltransferase ZDHHC13). ZDHHC is 660 AA with molecular weight of 70890 Da, it has 6 transmembrane domains and 6 ANK repeats (ANK 1–6), Zn_Fing (DHHC-TYPE) length is 51aa (from 426–476aa) and the Bompbias (Phe-rich) is 64aa (from 328–391aa). The active site (S-palmitoyl cysteine intermediate) is located at residue 456. Note that nonsense mutation arg-425-stop codon is located before both Zn_Fing and the active site of the protein. (0.10 MB TIF) [file pgen.1000985.s001.tif]
