# Supplementary material for: Mice with Alopecia, Osteoporosis, and Systemic Amyloidosis Due to Mutation in Zdhhc13, a Gene Coding for Palmitoyl Acyltransferase
Source: PLoS Genet. 2010 Jun 10;6(6):e1000985. doi: 10.1371/journal.pgen.1000985 (PMC2883605; doi:10.1371/journal.pgen.1000985)
Supplement: Table S1 — Peripheral blood complete blood counts in affected and wild-type mice. (0.05 MB RTF) [file pgen.1000985.s002.rtf]

Table S1. Peripheral blood complete blood counts in affected and wild-type mice.

Parameter  (1)	Adult group	Weaning  group	
	wild (n=6) (2)	mutant (n=6)  (2)	wild (n=5) (3)	mutant (n=5) (3)	
WBC   ×109/L	8.9±0.66	7.9±0.95	10.4±0.62	9.8±0.50	
Neutrophil   %	38.5±4.13	58.8±2.24 *	59.66±2.72	57.86±3.38	
Lymphocyte %	53.8±4.88	33.1±1.86 *	38.42±0.88	36.42±2.53	
Monocyte %	5.4±0.56	4.5±0.55	4.428±0.62	4.7±0.49 	
Eosinophil  %	1.6±0.12	1.1±0.21	1.744±0.22	1.314±0.32	
Basophil %	2±0.18	2.1±0.2	1.942±0.18	2.364±0.30	
RBC  ×1012/L	9.7±0.22	9.3±0.53	9.764±0.17	9.28±0.47	
Hemoglobin g/dl	15.3±0.16	15±0.67	14.8±0.48	14.6±0.63 	
Hematocrit (PCV) %	51±0.49	44.7±6.41	53.9±1.77	52.7±1.64	
MCV   fL	52.7±0.97	54.3±0.66	53.4±1.18	53.8±0.90 	
MCH   pg	15.8±0.26	16.2±0.24	14.86±0.65	15.06±0.43 	
MCHC  g/dl	23±0.15	29.7±0.13	27.44±0.93	27.84±1.06 	
RDW   %	20.4±1	20.2±0.47	21.98±0.33	21.44±0.47	
PLT   ×109/L	863±16.3	827±25.7	872.8±20.23	905.6±27.63	
MPV  fL	6.4±0.17	6.7±0.16	7.176±0.32	6.722±0.29	
PCT ×109/L 	0.5±0.02	0.63±0.05	0.5678±0.03	0.6504±0.08	
PDW  %	20.4±0.24	20.3±0.57	22±0.67	21.26±0.75	
(1) Values are (mean±SE), * P<0.05
(2)  Adult mice with an average age of 30±1.6 weeks.  (3)  Weaning mice with an age of 4 weeks.
