# Supplementary material for: Mice with Alopecia, Osteoporosis, and Systemic Amyloidosis Due to Mutation in Zdhhc13, a Gene Coding for Palmitoyl Acyltransferase
Source: PLoS Genet. 2010 Jun 10;6(6):e1000985. doi: 10.1371/journal.pgen.1000985 (PMC2883605; doi:10.1371/journal.pgen.1000985)
Supplement: Table S2 — Blood chemistry in affected and wild-type mice. (0.05 MB RTF) [file pgen.1000985.s003.rtf]

Table S2. Blood chemistry in affected and wild-type mice.

Parameter  (1)	Adult group	Weaning group	
	wild (n=6) (2)	mutant (n=6)  (2)	wild (n=5) (3)	mutant (n=5) (3)	
AST   (U/L)	52.8±5.07	100.6±15.3 *	56.4±3.09	101.6±15.6 *	
ALT    (U/L)	24.8±2.72	44.5±7.18 *	26.8±1.72	30.8±1.74	
CPK    U/L	15.9±7.9	13.0±37.3	31.6±5.24	39.2±4.87	
TCHO (mg/dl)	185±173.5	153.9±10.91	188.2±9.49	178±9.45	
TP      (g/dl)	4.6±0.2	5.2±0.28	4.8±0.16	4.68±0.28	
ALB     (g/dl)	2.95±0.06	3.04±0.07	2.56±0.13	2.8±0.23	
GLO    (g/dl)	1.65±0.17	2.13±0.26	2.24±0.11	1.88±0.42	
TBIL   (mg/dl)	0.5±0.06	0.69±0.06	0.44±0.03	0.39±0.02	
BUN   (mg/dl)	23.5±1.69	35.6±8.33	27.9±1.77	33.04±3.15	
Ca    (mg/dl)	6±0.79	5.21±0.4	5.38±0.79	6.24±0.82	
Mg    (mg/dl)	1.28±0.14	1.14±01	1.18±0.12	1.28±0.06	
CRP (mg/dl)	3.76±0.56 	2.35±0.35 (4)	3.63±0.47	2.49±0.39	

 (1) Values are (mean±SE), * P<0.05
(2)  Adult mice with an average age of 30±1.6 weeks.  (3)  Weaning mice with an age of 4 weeks.
(4)   Number of mutant adult mice are 39. 
